# Physical activity level among pregnant women attending maternal healthcare services in rural Karnataka; findings of a cross-sectional study

**DOI:** 10.12688/f1000research.151485.1

**Published:** 2024-10-14

**Authors:** Balamurugan Janakiraman, Arunachalam Ramachandran, Hariharasudhan Ravichandran, Kshama Susheel Shetty, Mohammad Sidiq, Aksh Chahal, T.S. Veeragoudhaman, Sathvik B Sridhar, Ramya Ramasamy Sanjeevi, Richa Hirendra Rai, Sonia Pawaria, Karthick Balasubramanian, Neha Kashyap, Krishna Reddy Vajrala, Mshari Alghadier

**Affiliations:** 1 Department of Physiotherapy, School of Allied Health Sciences, Madhav University, Abu Road, Sirohi, Rajasthan, 307026, India; 2 SRM College of Physiotherapy, Faculty of Medicine and Health Sciences, SRM Institute of Science and Technology, Kattankulathur, Tamil Nadu, 603203, India; 3 Alva's College of Physiotherapy & Research Centre, Alva's Education Foundation, Moodubudire, Dakshina Kannada, Karnataka, 574227, India; 4 Faculty of Physiotherapy, Meenakshi Academy of Higher Education and Research, Chennai, Tamil Nadu, 600 078, India; 5 Department of Physiotherapy, SAHS, Galgotias University, Greater Noida, Uttar Pradesh, 203201, India; 6 College of Pharmacy, RAK Medical and Health Sciences University, Ras Al Khaimah, 11172, United Arab Emirates; 7 Department of Physical Therapy, College of Nursing and Health Sciences, Jazan University, Jazan, Saudi Arabia; 8 School of Physiotherapy, Delhi Pharmaceutical Sciences and Research University, New Delhi, 110007, India; 9 Faculty of Physiotherapy, Shree Guru Gobind Singh Tricentenary University, Gurugram, Haryana, 122505, India; 10 Maharishi Markandeshwar Institute of Physiotherapy and Rehabilitation, Maharishi Markandeshwar, Mullana, Haryana, 133207, India; 11 Department of Health and Rehabilitation Sciences, College of Applied Medical Sciences,, Prince Sattam bin Abdulaziz University, Al Kharj, Riyadh Province, 16273, Saudi Arabia

**Keywords:** Pregnancy, Physical activity, Physical exercise, Physical activity pattern, Physical activity level

## Abstract

**Background:**

Regular exercise during pregnancy improves fetal and mother outcomes unless contraindicated. Despite being generally safe and beneficial, non-participation in prenatal activity is relatively common among most of the Asian countries due to multiple reasons. In India, findings related to maternal physical activity and its determinant are scant.

**Objective:**

The objective of this study is to assess the physical activity level and associated factors among pregnant women attending maternal healthcare services in Dakshina Kannada District in India.

**Method:**

A multi-center community-based cross-sectional study was conducted recruiting 424 pregnant women attending the maternal healthcare facilities at four taluks of Dakshina Kannada district in Karnataka state, India. A structured questionnaire that collected information on socio-demographic, and maternal characteristics was used and the Pregnancy Physical Activity Questionnaire tool was used to determine the physical activity during pregnancy. Logistic regression model was used to determine the predictor variables.

**Results:**

The prevalence of physical inactivity was 40.33%. Physical activity is favorable among pregnant women aged between 25 to 29 years, residing in an urban locality, diploma or graduation and being housewife. Determinants of physical inactivity during pregnancy were sedentary occupation (AOR 7.22, 95% CI 2.2, 23.4), low family income (AOR 3.16, 95% CI 1.414, 7.054), having one child (AOR 5.4, 95% CI 1.3, 22.2), during 2 ^nd^ trimester (AOR 2.513, 95% CI 1.5, 4.23) and self-reported lack of time (AOR 2.884, 95% CI 1.410, 5.901).

**Conclusion and recommendation:**

A moderate proportion of pregnant women reported physical inactivity during pregnancy in the Dakshina Kannada district, Karnataka. Physical inactivity was associated with sedentary employment, low income, number of children, trimester, and time constraints. Measures should be undertaken to promote the importance of recommended levels of physical activity, enhance access, and support system targeting pregnant women.

## Background

Physical activity is any bodily movement produced by skeletal muscles that requires energy expenditure, it includes domestic, work-related, transportation and recreational activities. ^1^ It plays a significant role in improving maternal and child health and prevention of chronic non-communicable diseases. ^2^ Physical activity during pregnancy has a significant role in preparation for labor effort and reduction of the duration of the labor. ^3^ It is also important to prevent pregnancy-related complications such as gestational diabetes mellitus, preeclampsia, gestational weight gain and improved maternal fitness and well-being, mood stability, decreased musculoskeletal discomfort and lower limb edema. ^4^^–^^11^ Not only for mothers it also benefits the fetus including decreased resting fetal heart rate, increased amniotic fluid level, improvement to the viability of the placenta, early neurobehavioral maturation and stress tolerance. ^4^^,^^11^^,^^12^ Additionally, it lowers the incidence of operative delivery and prematurity. ^4^

Pregnancy is a time of biological, social, psychological and behavioral changes associated with the decline of physical activity and exercise habits in pregnant women because of this most pregnant women especially in low and middle-income countries perceive rest and relaxation as being significantly more important than having regular exercise or being active during pregnancy. ^13^ The World Health Organization’s guideline on physical activity recommends that pregnant women engage in at least 150 minutes of moderate-intensity physical activity on most days/weeks in the absence of medical/obstetrical complications. But, high-risk activities like vigorous exercise and contact sports should be avoided. ^1^ Several developed countries like the United States, the United Kingdom, Canada, Australia, Denmark, and Japan have their physical activity guidelines for pregnant women. ^14^^–^^18^ In contrast, in developing countries like India the evidence base on implementing national physical activity guidelines is sparse.

Sustainable Development Goals for global development by 2030 have listed 17 goals with more than 100 indicators. Ensuring healthy lives and promoting well-being for all at all ages is the third goal listed under Sustainable Development Goals. In this goal, strategies to reduce global maternal mortality ratio < 70/100,000 live births, and to increase access to reproductive healthcare services by 2030 were recommended to national policymakers worldwide by the World Health Organization. Despite the standardized healthcare facilities and substantial progress made in contributing to Sustainable Development Goal 3 (SDG target 3.1) through a record decline in mortality rate from 26/1000 live births in 2019 to 21/1000 live births in 2020, the state of Karnataka is still facing challenges as least performer among the five southern states of India towards maternal mortality rate. According to a report by the president of the Karnataka State Obstetrics and Gynecological Association in 2022, the main reasons considered for the high maternal mortality rate are irregular medical checkups and non-adherence to medical advice. ^19^^–^^21^

Though the American College of Obstetricians and Gynecologists committee opinion number 804 recommends continuing physical activity during pregnancy and the postpartum period, globally, about 60% of women become inactive during pregnancy. ^14^ In India, the prevalence of physically active women during pregnancy ranges from 2.8% to 10.2% respectively. ^22^^,^^23^ Evidence in the literature insists on the necessity of physical activity for normal anatomic and physiologic changes in the growing fetus, the physical activity practice is commonly lacking in the general population. The physical activity dosages recommended based on obstetric evaluation have many positive effects on maternal and fetal health. However evidence reports that the majority of pregnant women tend to decrease their physical activity during pregnancy due to different factors. ^13^^,^^19^^,^^20^^,^^24^^,^^25^ Also sedentary lifestyle is reported as a major risk factor for 50% of Caesarean deliveries in Mangalore town, Karnataka. Therefore, the aim of this study is to assess the prevalence and associated factors of self-reported physical activity during pregnancy among pregnant women in Dakshina Kannada District of Karnataka.

## Methods

### Study design and period

This was a multi-center community-based cross-sectional descriptive study conducted among pregnant women in 8 maternal healthcare facilities in 4 taluks (administrative zones) of Dakshina Kannada district, situated in Karnataka state, India. This study was carried out from August 2023 to January 2024. The study protocol was approved by the institutional ethical review committee of the Alvas College of Physiotherapy and Research Centre (ref no; ACP/OP/CL/20230323/03, dated 23/03/2023). All the participants provided informed consent, and they adhered to the principles outlined in the Declaration of Helsinki. STROBE (Strengthening the Reporting of Observational Studies in Epidemiology) for cross-sectional reporting guidelines was used to assess the paper components of the manuscript.

### Study setting and participants

According to the 2011 census, Dakshina Kannada district has a population of 2.89 million of which 52.33% are rural habitants and 1.54 million are females. The district is divided into 9 taluks (administrative zones) and the gender-wise literacy was 93.13 and 84.13 for males and females respectively. The female literacy rate of the district was 79.8% in rural and 88.8% in urban zones. However, the female literacy rate of the study area (Dakshina Kannada) was higher than that of the Karnataka state which is 68.08. ^26^ The state is an achiever (100) for one of the 16 SDGs: affordable and clean energy; a performer (50–64) for six goals: zero hunger, quality education, gender equality, industry, innovation and infrastructure, climate action, life below water; and an aspirant (0–49) for none of the goals. Of the 16 SDGs, the state is an achiever (65–99) for nine of them: no poverty, good health and well-being, clean water and sanitation, decent work and economic growth, reduced inequality, sustainable cities and communities, sustainable consumption and production, life on land, peace, justice, and strong institutions. ^25^ According to the 2023 Niti Aayog report, the Dakshina Kannada district with 39 deaths per 1000 live births is far ahead of other districts. The NHFS-5 for the years 2019-2020 reported that the mother who had an antenatal check-up in their 1 ^st^ trimester and those who had at least 4 antenatal care visits were 86.1% and 82% respectively in the Dakshina Kannada district. As per the reports of the special bulletin by the Vital Statistics Division India for 2018-2020, the maternal mortality ratio (MMR) of Karnataka was 69 (95% CI 35, 103) compared to the MMR of 97 (95% CI 88, 106) of India.

### Population and eligibility criteria

All pregnant women aged 18 and above, Kannada speaking, native of the study area for the past 1 year, and those who attended the antenatal care clinic in those settings were included in this study. Pregnant women who presented with impaired verbal comprehension, visual impairment, or physical or psychological conditions that could interfere with comprehension and/or autonomy in consent to participate, mothers who were categorized as risk pregnancy and medically advised to avoid physical activity were excluded. All the participants signed the written informed consent and an impartial witness was present for mothers with no formal schooling (unable to read).

### Sample size and sampling technique

The study sample size was determined by using the single population proportion formula; by assuming a 5% level of significance, confidence interval of 95%, and 50% prevalence of physical inactivity less than the recommended guidelines by the pregnant women in the study area (non-availability of regional data). The derived power calculated sample was 384, the final sample size was n = 424 after accommodating for 15 % contingency plan. On average 30 to 35 pregnant women visit these maternal healthcare facilities (MHF) on average. To improve sample and time representativeness the data collection was limited to a maximum of ‘5’ in a single day for a single MHF. A multistage sampling technique was designed to attain the required power-calculated sample for this study.

**Figure 1.**
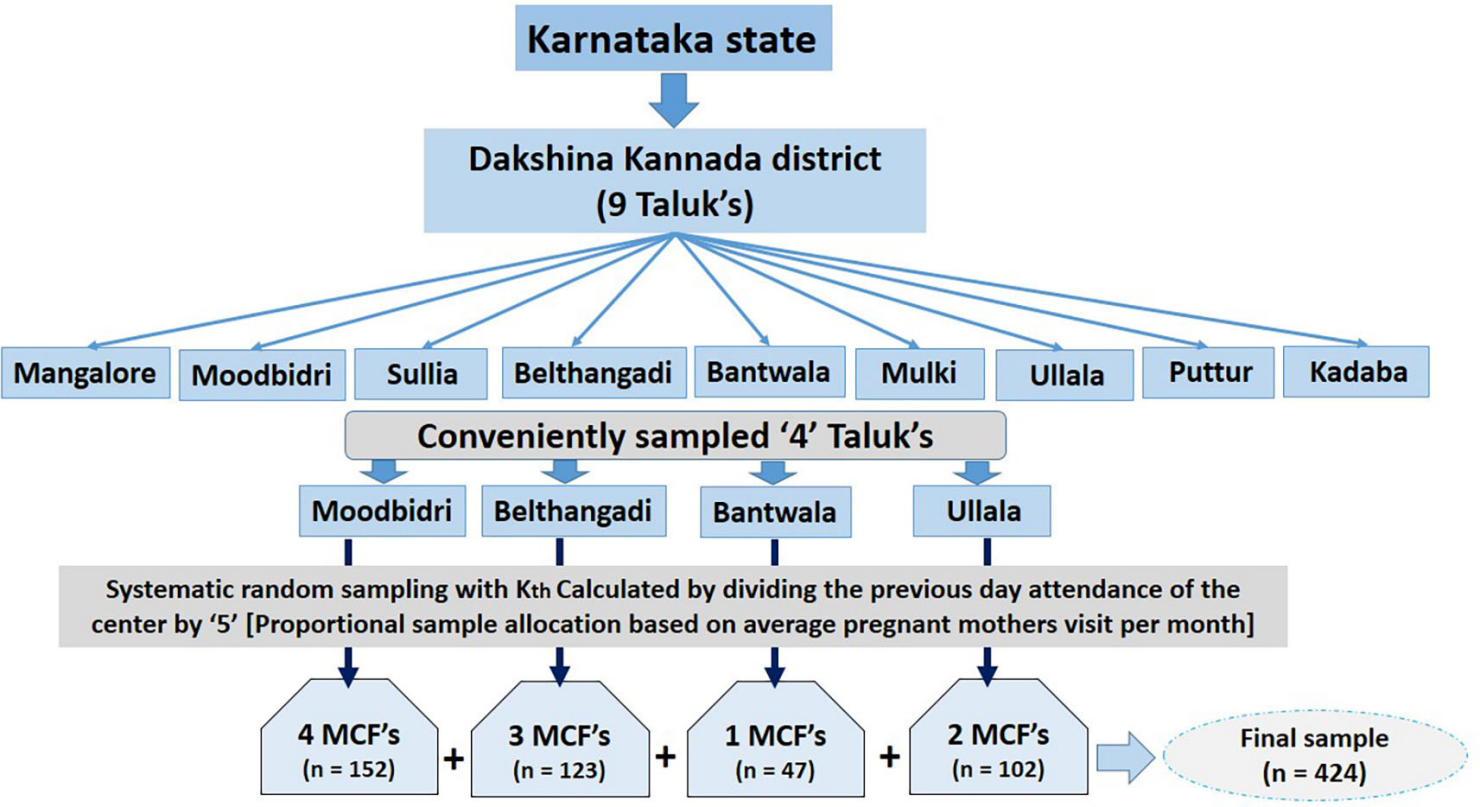
Sampling flow.

The Dakshina Kannada district has 2 revenue sub-divisions and nine taluks; Mangalore, Moodbidri, Mulki, Ullala, Bantawal, Puttur Belthangadi, Sullia, and Kadaba. We conveniently selected 4 taluks from where the Antenatal care clinics of outpatient Obstetrics and gynecology department of Alvas Health Center (Moodbidri), and the out-patient women’s health care department of Alvas Physiotherapy Clinic (Moodbidri) receive the majority of the clients. The MHF stationed at PHCs and Anganwadi centres were visited and that serves the majority of the women of that taluks were selected. Based on the previous year’s registry data, the required sample for each selected centre was proportionally allocated. So, during every data collection day, the Kath number was calculated by dividing the number of mothers who visited the center the previous working day by ‘5’ (maximum slab allowed). Data was collected until the proportional sample for the center was achieved Figure 1.

### Data collection tools and methods

Data were collected using a structured questionnaire through face-to-face interviews. Four Physiotherapy interns trained in data collection procedures were involved as the data collectors under the supervision of one Female Clinical Physiotherapist. The questionnaire was adapted from previous relevant literature. The structured questionnaire consisted of items related to socio-demographics, maternal characteristics, and pregnancy physical activity questionnaire tool. The pregnancy physical activity questionnaire was first developed in English ^24^ and then translated to the local language (Kannada) and then back to English to facilitate the understanding of the respondents. A one-day training was provided for the data collectors and a supervisor regarding the objectives of the study, way of approach to the participant, sampling techniques and procedures, client privacy issue, client confidentiality issue, client informed and voluntary participation, data collection method, and significance of the study. Then after, a pretest was conducted on 5% of the sample size before commencing the actual data collection. The pretest was performed at a private antenatal health care clinic, which is similar in culture and lifestyle to the main data collection setting. The purpose and objectives of the study were clearly stated on the first page of the questionnaire which the interviewers explained to participants. During data collection, there was close communication among the data collectors, a supervisor and a principal investigator. The collected questionnaires were checked for completeness and on spot corrective measures were taken both by data collectors and supervisors. Daily meetings have been conducted among the data collectors, a supervisor and a principal investigator for discussion regarding presenting difficulties and to assess the progress of data collection.

### Statistical analysis

Data were checked, coded and entered into Epi-info7 then exported to the SPSS version 23 software package for further analysis. Frequencies and cross-tabulations were used to summarize descriptive statistics. The data were presented by texts, tables and graphs. The overall proportion with 95% CI was calculated to determine the prevalence of moderate-intensity physical activity. Furthermore, bivariate logistic regression analysis was used to identify factors associated with moderate-intensity physical activity. Multivariate logistic regression was used to statistically adjust for covariates found to be statistically significantly associated with moderate physical activity at *p* < 0.05.

## Results

### Socio-demographic characteristics

A total of 424 questionnaires were distributed to pregnant women, all filled with a 96.9% response rate. The mean age of the participants was 26.89 ± 4.704 years. More than one-third of the participants, 37.5% (159) were between the age of 25-29 and most of them, 80.7% (342) were from urban areas. About 91.7% of the participants were married and nearly half of the participants were housewives. The other socio-demographic characteristics of the subjects are presented in Table 1.

**Table 1.** Socio-demographic characteristics of the pregnant women attending the ANC centers (n= 424).

Variables	n (%)	Active (253)	In-active (171)	χ ^2^	*p*
Age					
*18-24*	142(33.5)	70 (27.7)	72(42.1)	9.63	0.008
*25-29*	159(37.5)	102 (40.3)	57(33.3)		
*>30*	123(29)	81 (32.0)	42(24.6)		
Residence					
*Urban*	342(80.7)	195(77.1)	147(86)	5.17	0.023
*Rural*	82(19.3)	58(22.9)	24(14)		
Marital status					
*Married*	389(91.7)	235(92.9)	154(90.1)	1.8	0.407
*Single/not married*	16(3.8)	7(2.8)	9(5.3)		
*Divorced*	19(4.5)	11(4.3)	8(4.7)		
Educational status					
*No formal education*	46(10.8)	35(6.4)	11(13.8)	10.058	0.018
*Primary*	66(15.6)	43(17%)	23(13.5)		
*Secondary*	115(27.1)	58(22.9)	57(33.3)		
*Diploma and above*	197(46.5)	117(46.2)	80(46.8)		
Employment status					
*House wives*	208(49.1)	122(48.2)	86(50.3)	14.66	0.002
*Employed (Government/private)*	137(32.3)	83(32.8)	54(31.6)		
*Farmers*	53(12.5)	40(15.8)	13(7.6)		
*Own business*	26(6.1)	8(3.2)	18(10.5)		
Family monthly income (INR)					
*< 10000*	114(26.9)	79(31.2)	35(20.5)	9.73	0.02
*10000 to < 20,000*	102(24.1)	56(22.1)	46(26.9)		
*20,000 to 30,000*	108(25.5)	68(26.9)	40(23.4)		
*>30, 000*	100(23.6)	50(19.8)	50(29.2)		

### Maternal characteristics

More than one-third the participants were nulliparous 37.7% (160) and had no children and almost two-thirds of the participants were in the third trimester of their pregnancy ( Table 2).

**Table 2.** Maternal characteristics of the pregnant women attending the Antenatal health care centers (n = 424).

Variables	n (%)	Active	In-active	χ ^2^	*p*
Parity					
*0*	160(37.7)	71(28.1)	89(52)	28.84	0.000
*1*	70(16.5)	55(21.7)	15(8.8)		
*2*	94(22.2)	63(24.9)	31(18.1)		
≥ *3*	100(23.6)	64(25.3)	36(21.1)		
No of children					
*0*	163(38.4)	70(27.7)	93(54.4)	40.288	0.000
*1*	69(16.3)	57(22.5)	12(7)		
*2*	91(21.5)	66(26.1)	25(14.6)		
≥ *3*	101(23.8)	60(23.7)	41(24)		
Trimesters					
*1 ^st^*	21(5)	12(4.7)	9(5.3)	8.077	0.018
*2 ^nd^*	140(33)	97(38.3)	43(25.1)		
*3 ^rd^*	263(62)	144(56.9)	119(69.6)		

### Exercise-related characteristics

About 80.7% (342) participants had no regular physical activity before pregnancy and 79.7 % (338) pregnant women agreed that being active during pregnancy is important and only one-third of the participants had husbands who exercised regularly. Health institutions and families were the most commonly reported sources of information regarding the benefits of exercise during pregnancy and fear of miscarriage and too tired were the most reported reasons for not exercising during pregnancy ( Table 3).

**Table 3.** Exercise related characteristics of rural dwelling pregnant women attending Antenatal health care centers in Dakshina Kannada, Karnataka (n = 442).

Variables	n (%)	Active	In-active	χ ^2^	*p*
Regular exercise before pregnancy					
*Yes*	82(19.3)	57(22.5)	25(14.6)	4.092	0.043
*No*	342(80.7)	196(77.5)	146(85.4)		
Being active during pregnancy is important					
*Yes*	338(79.7)	206(81.1)	132(77.2)	1.13	0.23
*No*	86(20.3)	47(18.6)	39(22.8)		
Having husband exercise regularly					
*Yes*	165(38.9)	92(36.4)	73(42.7)	1.72	0.19
*No*	259(61.1)	161(63.6)			
**Source of information**					
Families					
*Yes*	258(60.8)	152(60.1)	106(62)	0.156	0.69
*No*	166(39.2)	101(39.9)	65(30.80		
Health institution					
*Yes*	273(64.4)	164(64.8)	109(63.7)	0.052	0.82
*No*	151(35.6)	89(35.2)	62(36.3)		
Media					
*Yes*	69(16.3)	40(15.8)	29(17)	0.099	0.75
*No*	355(83.7)	213(84.2)	142(83)		
**Barrier to exercise**					
Fear of miscarriage					
*Yes*	144(34)	83(32.8)	61(35.7)	0.374	0.54
*No*	280(66)	170(67.2)	110(64.3)		
Discomfort					
*Yes*	39(9.2)	25(9.9)	14(8.2)	0.35	0.554
*No*	385(90.8)	228(90.1)	157(91.8)		
Lack of exercise knowledge					
*Yes*	95(22.4)	68(26.9)	27(15.8)	7.216	0.007
*No*	329(77.6)	185(73.1)	144(84.2)		
Too tired					
*Yes*	120 (28.3)	68(26.9)	52(30.4)	0.627	0.428
*No*	304 (71.7)	185(73.10	119(69.6)		
Having no time					
*Yes*	60(14.2)	31(12.3)	29(17)	1.86	0.173
*No*	364(85.8)	222(87.7)	142(83)		
Weight gain					
*Yes*	18(4.2)	9(3.6)	9(5.3)	0.73	0.4
*No*	406(95.8)	244(96.4)	162(94.7)		

### Physical activity demonstrated in each trimesters

The median physical activity was marginally raised in third trimester [3.38 (1.29)] compared to 1 ^st^ [2.63 (0.989)] and 2 ^nd^ trimester [2.39 (2.84)], respectively ( Table 4). The median frequency obtained for light intensity activity across all the trimesters were similar (3.3 vs 3.5 vs 3.5), respectively. The amount of energy spent on occupational activities diminished as the trimester progressed (2.45 vs 1.75 vs 0.88) and the energy spent by women in household activities did not differ across the trimesters (2.45 vs 2.52 vs 2.52).

**Table 4.** Physical activities demonstrated across each trimester in women attending Antenatal health care.

Variables	1 ^st^ trimester	2 ^nd^ trimester	3 ^rd^ trimester
	Median (variance)	Median (variance)	Median (variance)
Total energy expenditure/week (MET-hours/week)	2.63(0.989)	2.39(2.84)	3.38(1.29)
Based on intensity of activity			
Sedentary	5.95(12.2)	4.9(18.86)	4.9(16.550
Light	3.3(3.16)	3.5(6.6)	3.5(4.64)
Moderate	0.54(0.75)	0.81(2.12)	0.56(1.2)
Vigorous	0.00(0.00)	0.00(0.00)	0.00(0.00)
Based on type of activity			
Household	2.45(3.54)	2.525(3.436)	2.525(3.286)
Occupational	2.45(19.048)	1.75(37.36)	0.88(22.50)

### Factors associated with physical activity

All the independent variables were included in the bivariate analysis with a cut-off significance of P < 0.25 for further final model multivariate analysis. The bivariate analysis results ( Table 5) demonstrated the following independent variables as age, residence, educational status, work status, family monthly income, parity, number of children, trimesters, regular exercise before pregnancy, lack of exercise knowledge and having no time had P value < 0.25 and were included for multivariate analysis. The multivariable regression analysis results identified several independent variables that are associated with the physical activity of pregnant women.

**Table 5.** Bivariate logistic regression of independent variables associated with physical activity of pregnant women.

Variables	n (%)	Active	Inactive	COR	*95% CI*	*p*
Age						
*18-24*	142(33.5)	70	72(42.1)	1 ref		
*25-29*	159(37.5)	102	57(33.3)	1.84	1.16, 2.92	0.01
*>30*	123(29)	81	42(24.6)	1.98	1.21, 3.26	0.007
Residence						
*Urban*	342(80.7)	195(77.1)	147(86)	1 ref		
*Rural*	82(19.3)	58(22.9)	24(14)	1.82	1.08, 3.07	.024
Marital status						
*Married*	389(91.7)	235(92.9)	154(90.1)	1.11	0.437, 2.82	0.83
*Divorce/Widow*	16(3.8)	7(2.8)	9(5.3)	0.56	0.15, 2.17	0.4
*Cohabitat*	19(4.5)	11(4.3)	8(4.7)	1 ref		
Educational status						
*No formal education*	46(10.8)	35(6.4)	11(13.8)	2.18	1.04, 4.54	0.04
*Primary*	66(15.6)	43(17)	23(13.5)	1.28	0.72, 2.29	0.41
*Secondary*	115(27.1)	58(22.9)	57(33.3)	0.70	0.44, 1.11	0.12
*Diploma and above*	197(46.5)	117(46.2)	80(46.8)	1 ref		
Work status						
*Housewife*	208(49.1)	122(48.2)	86(50.3)	3.192	1.327, 7.68	0.01
*Civil servant*	137(32.3)	83(32.8)	54(31.6)	3.458	1.405, 8.5	0.007
*Merchant*	53(12.5)	40(15.8)	13(7.6)	6.923	2.443, 19.6	0.000
*Student*	26(6.1)	8(3.2)	18(10.5)	1 ref		
Family income/month						
*< 10000*	114(26.9)	79(31.2)	35(20.5)	2.257	1.291, 3.946	0.004
*10000 to < 20,000*	102(24.1)	56(22.1)	46(26.9)	1.217	0.7, 2.12	0.486
*20,000 to 30,000*	108(25.5)	68(26.9)	40(23.4)	1.700	0.978, 2.956	0.06
*>30, 000*	100(23.6)	50(19.8)	50(29.2)	1 ref		
Parity						
*0*	160(37.7)	71(28.1)	89(52)	0.45	0.27, 0.75	0.002
*1*	70(16.5)	55(21.7)	15(8.8)	2.06	1.022, 4.16	0.043
*2*	94(22.2)	63(24.9)	31(18.1)	1.143	0.632, 2.07	0.66
≥ *3*	100(23.6)	64(25.3)	36(21.1)	1 ref		
Number of children						
*0*	163(38.4)	70(27.7)	93(54.4)	0.514	0.31, 0.85	0.01
*1*	69(16.3)	57(22.5)	12(7)	3.246	1.55, 6.792	0.002
*2*	91(21.5)	66(26.1)	25(14.6)	1.8	0.98, 3.3	0.056
≥ *3*	101(23.8)	60(23.7)	41(24)	1 ref		
Trimester						
*1 ^st^*	21(5)	12(4.7)	9(5.3)	1.102	0.45, 2.7	0.832
*2 ^nd^*	140(33)	97(38.3)	43(25.1)	1.864	1.208, 2.876	0.005
*3 ^rd^*	263(62)	144(56.9)	119(69.6)	1 ref		
Regular exercise before pregnancy						
*Yes*	82(19.3)	57(22.5)	25(14.6)	1 ref		
*No*	342(80.7	196(77.5)	146(85.4)	0.56	0.351, 0.987	0.045
Being active during pregnancy is important						
*Yes*	338(79.7)	206(81.1)	132(77.2)	1 ref		
*No*	86(20.3)	47(18.6)	39(22.8)	0.772	0.48, 1.25	0.29
Regular exercise of spouse						
*Yes*	165(38.9)	92(36.4)	73(42.7)	Ref		
*No*	259(61.1)	161(63.6)	98(57.3)	0.77	0.52, 1.14	0.19
**Source of information**						
*Families*						
*Yes*	258(60.8)	152(60.1)	106(62)	Ref		
*No*	166(39.2)	101(39.9)	65(30.80	1.08	0.73, 1.6	0.69
*Health institution*						
*Yes*	273(64.4)	164(64.8)	109(63.7)	Ref		
*No*	151(35.6)	89(35.2)	62(36.3)	0.954	0.64, 1.43	0.8
*Media*						
*Yes*	69(16.3)	40(15.8)	29(17)	Ref		
*No*	355(83.7)	213(84.2)	142(83)	1.087	0.65,1.84	0.75
**Barrier to exercise**						
*Fear of miscarriage*						
*Yes*	144(34)	83(32.8)	61(35.7)	Ref		
*No*	280(66)	170(67.2)	110(64.3)	1.14	0.76, 1.7	0.54
*Discomfort*						
*Yes*	39(9.2)	25(9.9)	14(8.2)	Ref		
*No*	385(90.8)	228(90.1)	157(91.8)	0.813	0.4, 1.6	0.55
Lack of knowledge about exercise						
*Yes*	95(22.4)	68(26.9)	27(15.8)	Ref		
*No*	329(77.6)	185(73.1)	144(84.2)	0.5	0.31, 0.84	0.008
Too tired						
*Yes*	120 (28.3)	68(26.9)	52(30.4)	Ref		
*No*	304 (71.7)	185(73.10)	119(69.6)	0.84	0.55, 1.29	0.43
Having no time						
*Yes*	60(14.2)	31(12.3)	29(17)	Ref		
*No*	364(85.8)	222(87.7)	142(83)	1.5	0.85, 2.5	0.17
Weight gain						
*Yes*	18(4.2)	9(3.6)	9(5.3)	1 ref		
*No*	406(95.8)	244(96.4)	162(94.7)	1.5	0.585, 3.875	0.4

P < 0.25 is considered significant cut off for multivariate analysis.

Women merchants (selling goods, online retailers, business) are 7.2 times more likely to be sedentary (95% CI: 2.2 to 23.4) than those employed in civil services and housewives. Pregnant women with lower family income per month demonstrated 3.16 times more sedentary lifestyles (95% CI 1.414 to 7.054) compared to the higher income groups. Pregnant women who had 1 child were 5.4 times more sedentary (95% CI: 1.3 to 22.2) than those who had 2 children. It is also revealed that pregnant women in the first trimester were least sedentary compared to those in the second and third trimesters. Pregnant women with no participation in regular physical activity before pregnancy were 2.39 times protected from being sedentary during pregnancy. Pregnant women lacking time to be active were 2.884 times sedentary compared with those who had time ( Table 6).

**Table 6.** Bivariate and multivariable logistic regression analysis result of physical activity and associated factors during pregnancy among pregnant women.

Variables	n (%)	Active	In-active	*AOR*	*95% CI*	*p*
Work status						
*Housewife*	*208(49.1)*	122(48.2)	86(50.3)	1.24	0.44, 3.5	0.68
*Civil servant*	137(32.3)	83(32.8)	54(31.6)	2.4	0.82, 7.011	0.111
*Merchant*	53(12.5)	40(15.8)	13(7.6)	**7.2**	**2.2, 23.4**	**0.001**
*Student*	26(6.1)	8(3.2)	18(10.5)	1 ref		
Family income/month						
*< 10000*	102(24.1)	56(22.1)	46(26.9)	3.16	1.414, 7.054	0.005
*10000 to < 20,000*	108(25.5)	68(26.9)	40(23.4)	1.634	0.81, 3.3	0.171
*20,000 to 30,000*	100(23.6)	50(19.8)	50(29.2)	2.531	1.28, 5	0.008
*> 30, 000*	208(49.1)	122(48.2)	86(50.3)	1 ref		
No of children						
*0*	163(38.4)	70(27.7)	93(54.4)	1.371	0.88, 3.97	0.999
*1*	69(16.3)	57(22.5)	12(7)	5.4	1.3, 22.2	0.02
*2*	91(21.5)	66(26.1)	25(14.6)	3.0	1.084, 8.3	0.034
≥ *3*	101(23.8)	60(23.7)	41(24)	1 ref		
Trimesters						
*1 ^st^*	21(5)	12(4.7)	9(5.3)	0.82	0.26, 2.6	0.736
*2 ^nd^*	140(33)	97(38.3)	43(25.1)	2.513	1.5, 4.23	0.001
*3 ^rd^*	263(62)	144(56.9)	119(69.6)	1 ref		
Regular exercise before pregnancy						
*Yes*	82(19.3)	57(22.5)	25(14.6)	Ref		
*No*	342(80.7	196(77.5)	146(85.4)	0.418	0.216, 1.808	0.08
Having no time						
*Yes*	60(14.2)	31(12.3)	29(17)	1 ref		
*No*	364(85.8)	222(87.7)	142(83)	2.884	1.410, 5.901	0.004

## Discussion

The purpose of this cross-sectional study is to determine the level of physical activity and determinants of the sedentary lifestyle during pregnancy among women attending antenatal health care centers in Dakshina Kannada district of Karnataka, India. The study findings revealed that 40.33% of women were physically inactive and 59.7% self-reported to practice the recommended level of physical activity. Overall, a moderate proportion of pregnant mothers reported below-par physical levels in the study area.

The prevalence of physical inactivity found in this study is higher than the prevalence reported in the Bangalore (India) study 7.2% in 2021, ^27^ Zonal hospitals of Tigray (Ethiopia) 21.9% in 2019, ^28^ Serbia 27.2% in 2020, ^29^ Northern Sweden 27.3% in 2021, ^30^ and Riyadh (Saudi Arabia) 41.62% in 2020. ^31^ At the same time, the prevalence is lesser than the reported prevalence among the Western pregnant women population (75%) in 2016, ^32^ pregnant women attending the Institutional antenatal care facility of Tigray (Ethiopia) (79.3%) in 2020, ^28^ Middle eastern pregnant women (84%) in 2016, ^30^ and pregnant women in South Asia (86%) in 2016. ^32^ The differences in the level of literacy, economic background, awareness and accessibility towards antenatal health education and care, socio-cultural practices and beliefs, lifestyle practices and development in advanced technologies could be contributing factors for the varying range of prevalence worldwide. The second objective of this study is to identify the factors associated with a sedentary lifestyle during pregnancy. Type of employment, family income, number of children, stage of trimester, regular exercise before pregnancy and lack of time were the factors identified as significantly associated with physical inactivity among pregnant women attending antenatal care in Dakshina Kannada, Karnataka. Pregnant women involved in occupations such as merchants or businesses lack physical activity seven times compared to women involved in civil services and household activities. Women occupied as merchants or owned self-business were involved in prolonged sitting. They were lacking both occupational and leisure time physical activity. Involvement in sedentary occupation could also predispose pregnant women to adverse effects such as gestational diabetes, gestational hypertension, gestational weight gain and so on. ^33^ At the same time excessive physical exertion at any occupation such as trunk forward bending, rotation, reaching and lifting could increase intra-abdominal pressure and can result in miscarriage or spontaneous abortion. ^34^ This insists on the need for educating pregnant women about the safer level of physical exertion that could be accomplished in the workplace. In the present study, the pregnant women with low family income were three times physically inactive compared to average and high family income categories. Previous studies ^35^^–^^37^ in the literature have reported that pregnant women with low family income were physically active compared to average and high income. The population included in those studies differs from the present study by socio-cultural aspects, financial background and lifestyle practices. Both state and central governments of India have implemented antenatal care and nutritional schemes such as Mathrushree, Mathrupoorna, Accredited Social Health Activist - ASHA package, Samagra Mathru Arogya Palana and so on for pregnant women below the poverty line. Though these schemes provide nutritional and financial assistance for antenatal care, there is no clear data to identify the reach of the American College of Obstetricians and Gynecologists’ recommended guidelines for physical activity in pregnant women from low family income. Lack of awareness on physical activity guidelines could be a contributing factor for physical inactivity among pregnant women with low family income. This necessitates the need for the promotion and implementation of physical activity in pregnant women with low family income living in south India.

The study findings identified that pregnant women with one child were five times physically active compared to 2 children and no children. The study finding is inconsistent with the previous reports from Ethiopia ^28^^,^^38^ and Nigeria. ^39^ In those studies, ^28^^,^^38^^,^^39^ moderate physical activity is reported in pregnant women parenting two or more children. The higher rate of fertility and high magnitude of multiparity exists among the Ethiopian and Nigerian populations and which is average or low among the population included in this study. This could be a contributing reason for differences in physical activity patterns of parenting pregnant women. The parenting responsibilities differ in pregnant women with younger and elder children; and in primiparous and multiparous situations. Pregnant women with one young child are in need to deliver more parental responsibilities compared to pregnant women with two or three elder children and hence lack time for participating in physical activities. Probably the nulliparous pregnant women could able to find time to perform physical activity and hence they are not sedentary compared to pregnant women with one child. In this study, it is observed that physical activity declines in each trimester. Pregnant women in the second trimester were two times physically inactive compared to the first trimester. Lack of adequate knowledge and practice towards programming physical activity in each trimester is prevailing commonly in pregnant women. Additionally, fear of falling, miscarriage/spontaneous abortion, and anxiety refrains them from participating in physical activity programs during pregnancy. This is attributed to the trend of declining physical activity in advanced trimesters among pregnant women in Dakshina Kannada district. This study also revealed that pregnant women lacking schedules were two times physically inactive compared to those who were able to manage their time. More time spent in household or family activities and occupation could have compromised the time spent involvement in physical activity programs.

There were a few limitations in this present cross-sectional study. Firstly the collected data were self-reported and hence might have been affected by potential reporting bias. Secondly, there could be varied perceptions towards physical activity programming guidelines among the participants and lastly, the collected data lacking objective measurements. Nevertheless, these power-calculated sampled study reports valuable insights into the variables that are determinants of the physical activity levels among pregnant mothers in the study area. Further, the sampling procedure used and data collection strategy to limit the recording to five respondents per day per center would have enhanced the time and sample representation in this study.

## Conclusion

This study found that a moderate proportion of pregnant mothers were not engaged in physical activity to the levels recommended by the ACOG guidelines. The determinants that negatively influenced physical activity were low family income, sedentary occupation, being primiparous, mothers in their second trimester, and reporting of lack of time as the reason.

Though the national policies and schemes are favorably implemented towards the promotion of safer antenatal care, there still exists a deficiency in knowledge and practice towards the recommended level of physical activity guidelines in pregnancy. Strategies towards the target population are warranted to improve the physical activity status and to sensitize the benefits of being active during pregnancy.

## Ethics and consent

The study protocol was approved by the institutional ethical review committee of the Alvas College of Physiotherapy and Research Centre (ref no; ACP/OP/CL/20230323/03) dated 22-03-2023. All the participants provided written informed consent, and they adhered to the principles outlined in the Declaration of Helsinki.

## Data Availability

The dataset contains comprehensive data collected from a study that examines physical activity levels among pregnant women across different trimesters and varying education levels. The dataset is structured to facilitate analysis of the relationship between pregnancy stages (trimesters), physical activity patterns, and educational backgrounds. ^40^ Repository: Figshare **Title of Project**: **Physical activity level among pregnant women attending maternal healthcare services in rural Karnataka; findings of a cross-sectional study** DOI: https://doi.org/10.6084/m9.figshare.25488730.v1 This project contains the following underlying data:
1. File Name: Physical Activity Dataset 2. Code Book of Physical Activity Dataset Data are available under the terms of the Creative Commons Attribution 4.0 International license (CC-BY 4.0).
