# Supplementary material for: Physical activity level among pregnant women attending maternal healthcare services in rural Karnataka; findings of a cross-sectional study
Source: F1000Res. 2026 Feb 27;13:1223. Originally published 2024 Oct 14. [Version 2] doi: 10.12688/f1000research.151485.2 (PMC13049419; doi:10.12688/f1000research.151485.2)
Supplement: Peer review history

# Reviewer report: Reviewer response for version 2

**DOI:** 10.5256/f1000research.174768.r468225

**Published:** 2026-04-03

**Authors:** Dewi Marfuah

**Affiliations:** 1 PPNI West Java College of Nursing, West Java, Indonesia

Overall, the manuscript is well-written and generally suitable for Indexing.

 However, the literature review should be strengthened by incorporating more recent references.

 It is recommended to update the bibliography with studies from the last five years and replace outdated sources to ensure alignment with the latest scientific evidence.

 This will enhance the manuscript’s relevance, improve its scientific rigor, and demonstrate engagement with current developments in the field, particularly in rapidly evolving topics such as COVID-19 and maternal health.

Is the work clearly and accurately presented and does it cite the current literature?

Partly

If applicable, is the statistical analysis and its interpretation appropriate?

Yes

Are all the source data underlying the results available to ensure full reproducibility?

Yes

Is the study design appropriate and is the work technically sound?

Yes

Are the conclusions drawn adequately supported by the results?

Yes

Are sufficient details of methods and analysis provided to allow replication by others?

Yes

Reviewer Expertise:

Pregnant women, Maternity Nursing

I confirm that I have read this submission and believe that I have an appropriate level of expertise to confirm that it is of an acceptable scientific standard.

# Reviewer report: Reviewer response for version 1

**DOI:** 10.5256/f1000research.166132.r332084

**Published:** 2024-10-31

**Authors:** Dhruv Taneja, Prof. Dr. Waribam Ranjeeta

**Affiliations:** 1 Professor: Jaipur Physiotherapy College, Maharaj Vinayak Global University, Jaipur, Rajasthan, India; 2 Assistant Professor at Pacific College of Physiotherapy, Pacific Medical University, Udaipur, Rajasthan, India

Report: The study is well drafted with complete chapters in detail and the statistical analysis is upto the mark with well labelled figures into it. Only thing which needs to be documented is the future scope and recommendations needs to be included for future researchers. It meets the requirements to be indexed.

 Thank You

Is the work clearly and accurately presented and does it cite the current literature?

Yes

If applicable, is the statistical analysis and its interpretation appropriate?

Yes

Are all the source data underlying the results available to ensure full reproducibility?

Yes

Is the study design appropriate and is the work technically sound?

Yes

Are the conclusions drawn adequately supported by the results?

Yes

Are sufficient details of methods and analysis provided to allow replication by others?

Yes

Reviewer Expertise:

Physical Therapy, rehabilitation, Musculoskeletal and Sports Therapy, cardiothoracic rehabilitation, Neurological Rehabilitation

We confirm that we have read this submission and believe that we have an appropriate level of expertise to confirm that it is of an acceptable scientific standard.

# Response

**Published:** 2024-10-31

**Authors:** Dr Mohammad Sidiq

Thank you so much learner reviewers for your expert comment. We will surely instill your recommendations in our updated manuscript.

 Best regards
